# Inactivation of M2 AChR/NF-κB signaling axis reverses epithelial-mesenchymal transition (EMT) and suppresses migration and invasion in non-small cell lung cancer (NSCLC)

**DOI:** 10.18632/oncotarget.5004

**Published:** 2015-08-20

**Authors:** Qingnan Zhao, Jinnan Yue, Chun Zhang, Xiajing Gu, Hongzhuan Chen, Lu Xu

**Affiliations:** ^1^ Department of Pharmacology, Shanghai Jiao Tong University School of Medicine, Shanghai, China

**Keywords:** non-neuronal cholinergic system, NSCLC, EMT, NF-κB, M2 muscarinic receptor

## Abstract

Non-neuronal cholinergic system is involved in lung physiology and lung cancer. However, the biochemical events downstream acetylcholine (ACh) receptor activation leading to carcinogenesis and tumor progression are not fully understood. Our previous work has shown that non-neuronal ACh acts as an autoparacrine growth factor to stimulate cell proliferation and promote epithelial-mesenchymal transition (EMT) in non-small cell lung cancer (NSCLC) via activation of M2 muscarinic receptor (M2R). The aim of the present study was to delineate the underlying mechanisms linking M2R and lung tumor progression, which may provide potential therapeutic targets to delay lung cancer progression. Inhibition of M2R by antagonist or siRNA suppresses NSCLC cell migratory and invasive capacities, reverses EMT and simultaneously inactivates PI3K/Akt, MAPK ERK and NF-κB p65. On the other hand, M2R activation stimulates NSCLC migration and invasion and promotes EMT via NF-κB p65 activation. Moreover, NF-κB p65 activation induced by M2R activation was partially inhibited by either Akt or ERK inhibitor. Taken together, these results demonstrated for the first time that NF-κB p65 activation is essential in NSCLC progression associated with non-neuronal cholinergic system. Our data suggest that M2R/ERK/Akt/NF-κB axis could be a potential target for NSCLC treatment.

## INTRODUCTION

Acetylcholine (ACh) is an important neurotransmitter whose effects are mediated by nicotinic acetylcholine receptors (nAChR) and muscarinic acetylcholine receptors (mAChR). Growing evidence has shown that various non-neuronal cells, such as airway epithelial cells and lung cancer cells can synthesize and release ACh. Non-neuronal ACh is believed to act as an autoparacrine growth factor through activation of nAChR and mAChR [1–4]. Our and many other studies have demonstrated that markedly upregulated non-neuronal cholinergic system in lung cancer could be targeted for therapeutic intervention [5–10].

Most lung cancer patients are diagnosed at a locally advanced or metastatic stage with a dismal five-year survival rate. Epithelial-mesenchymal transition (EMT), which was first recognized as critical in embryogenesis, has been implicated in tumor progression and metastasis [11]. EMT can be triggered by growth factors including epidermal growth factor (EGF), hepatocyte growth factor (HGF) and fibroblast growth factor (FGF), as well as transforming growth factor β (TGFβ) and their downstream signaling pathways often involving PI3K/Akt and MAPK ERK pathways [12]. Our previous studies have shown that non-neuronal ACh, as an autoparacrine growth factor, induces EMT in NSCLC cells partially through activation of M2 muscarinic receptor (M2R) and blocking M2R signaling reverses EMT under basal and AChR agonist-induced conditions [10]. Activation of nAChR by nicotine has also been reported to induce EMT in lung and other cancer cells [13–15]. Those findings have indicated that non-neuronal cholinergic system could be involved in the progression of lung cancer through regulation of EMT. A hallmark of EMT is the loss of E-cadherin expression, which is mainly mediated by several transcription factors referred to as EMT master regulators including the Snail, Zeb and Twist families. The expression of these EMT master regulators is modulated by numerous signaling molecules, including nuclear factor κB (NF-κB) [12,16–18].

The NF-κB family has five proteins: RelA (p65), RelB, Rel, NF-κB1 (p50/p105), and NF-κB2 (p52/p100), each of which may form homo- or heterodimers. The activated NF-κB exists primarily as a heterodimer of p65/p50 or p52/RelB. In most cell types, NF-κB is sequestered in the cytoplasm in an inactive state by inhibitor of κB-alpha (IκBα). In the presence of activating signals, IκBα is phosphorylated by IκB kinase (IKK) leading to its degradation. NF-κB released from IκBα translocates to the nucleus and regulates the transcription of target genes by binding to specific DNA-binding sites. Besides the degradation of IκBα and nuclear translocation of NF-κB dimers, optimal activation of NF-κB also requires phosphorylation of NF-κB by a variety of kinases [19, 20]. NF-κB plays critical roles in the processes of development and progression of cancers [21]. NF-κB has been reported to be constitutively activated in most human cancers, but not in normal tissues [22]. Constitutively activated NF-κB is also tightly related to the tumor metastasis [23, 24]. Moreover, evidence has indicated that NF-κB activation is required for the induction and maintenance of EMT [25]. NF-κB activation has been found to upregulate the expression of EMT master regulators including Snail, Slug, Twist1 and ZEB1/2 to induce EMT [26–31].

In our previous study, we have shown that EMT could be induced by non-neuronal ACh, secreted by NSCLC cells partially through activation of M2 muscarinic receptors (M2R) [10]. The goal of this study is to delineate the underlying molecular mechanisms. Here we showed that antagonizing M2R resulted in the inactivation of NF-κB p65, PI3K/Akt and MAPK ERK as well as the reversal of EMT and reduced migratory and invasive capacities in NSCLC. On the other hand, muscarinic activation by pilocarpine stimulated NSCLC migration and invasion, promoted EMT and activated NF-κB p65 signaling, all of which was ablated by specific NF-κB inhibitor or p65 siRNA. Moreover, pilocarpine-induced NF-κB p65 activation was partially inhibited by either Akt or ERK inhibitor. Collectively, our results supported the proposal that non-neuronal ACh may activate M2R and downstream ERK and Akt to enhance NF-κB signaling, which promotes EMT and increases migratory and invasive ability in NSCLC. To the best of our knowledge, this is the first study to demonstrate that M2R/ERK/Akt/NF-κB signaling axis plays an important role in lung cancer progression involving non-neuronal cholinergic system. Our study may provide potential therapeutic targets to delay lung cancer progression.

## RESULTS

### Blocking M2R signaling suppressed NSCLC cell migration and invasion

**Figure 1:**
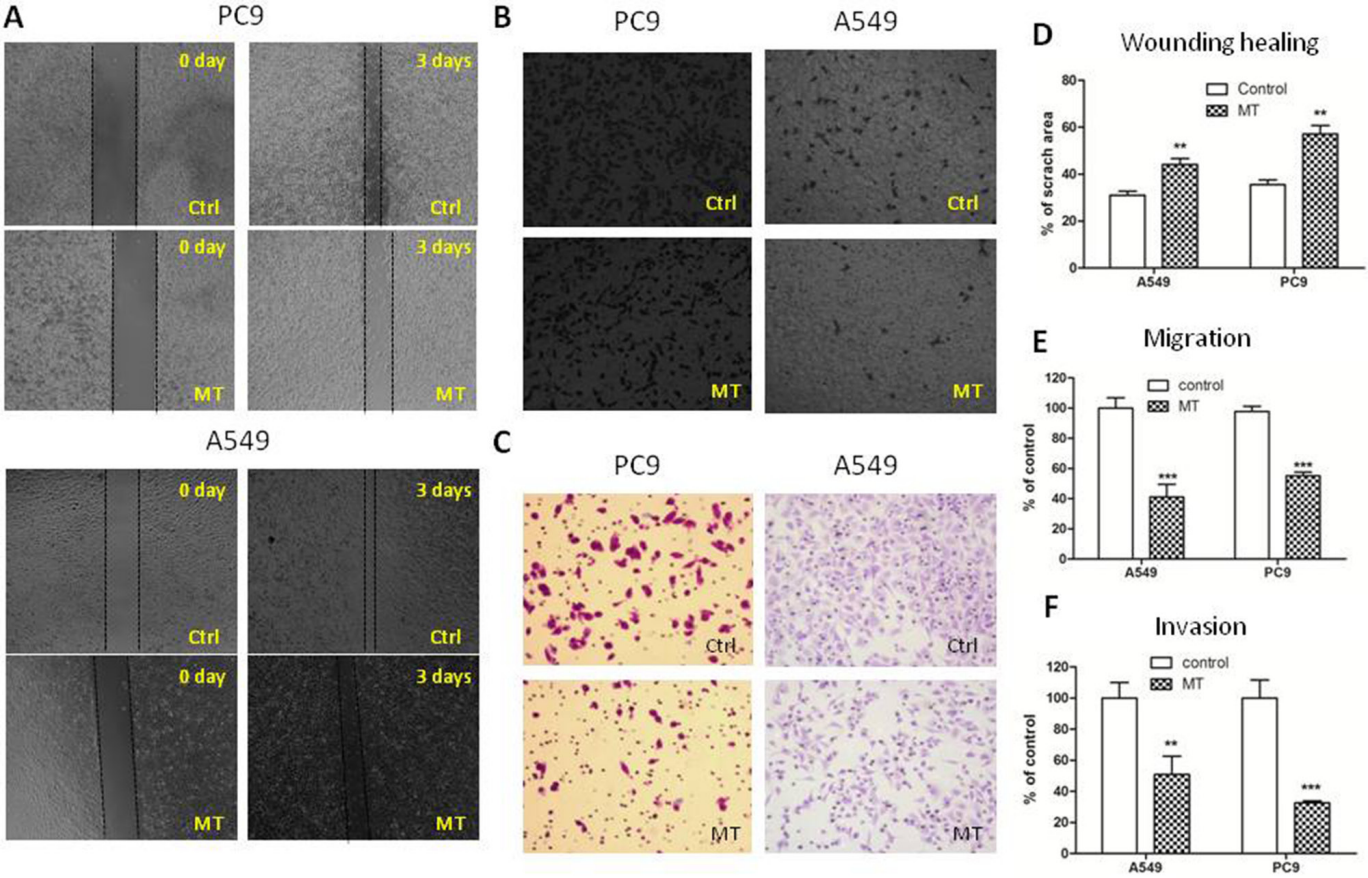
M2R antagonist methoctramine suppresses the migration and invasion of NSCLC cells **A.** Wound healing assay of A549 and PC9 cells treated with methoctramine (MT). After being scratched with a 100 μL pipette tip, cells were treated with 1 μM MT for 72 h. Images were taken at 0 and 72 h with a 4× objective lens. The black dotted lines depict the area of wound closure. Transwell migration **B.** and invasion **C.** assay of A549 and PC9 cells treated with MT. After 8 h (B) or 24 h (C) of incubation in presence or absence of 1 μM MT, cells remaining above the insert membrane were removed by gentle scraping with a sterile cotton swab. Cells that traveled through the insert membrane without (B) or with (C) Matrigel were fixed, stained with crystal violet and counted under light microscopy. Images were taken with a 10× objective lens. **D.** Quantification of wound healing assay. The open wound area was quantified by ImageJ from at least four independent microscopic fields and normalized to the area at time 0. The data are presented as the mean ± SEM. Quantification of transwell migration **E.** and invasion **F.** assay. The number of cells was counted from at least four independent microscopic fields. The data are presented as the means ± SEM and normalized to cells treated with solvent (DMSO). *P < 0.05; **P < 0.01; ***P < 0.001, compared with control.

**Figure 2:**
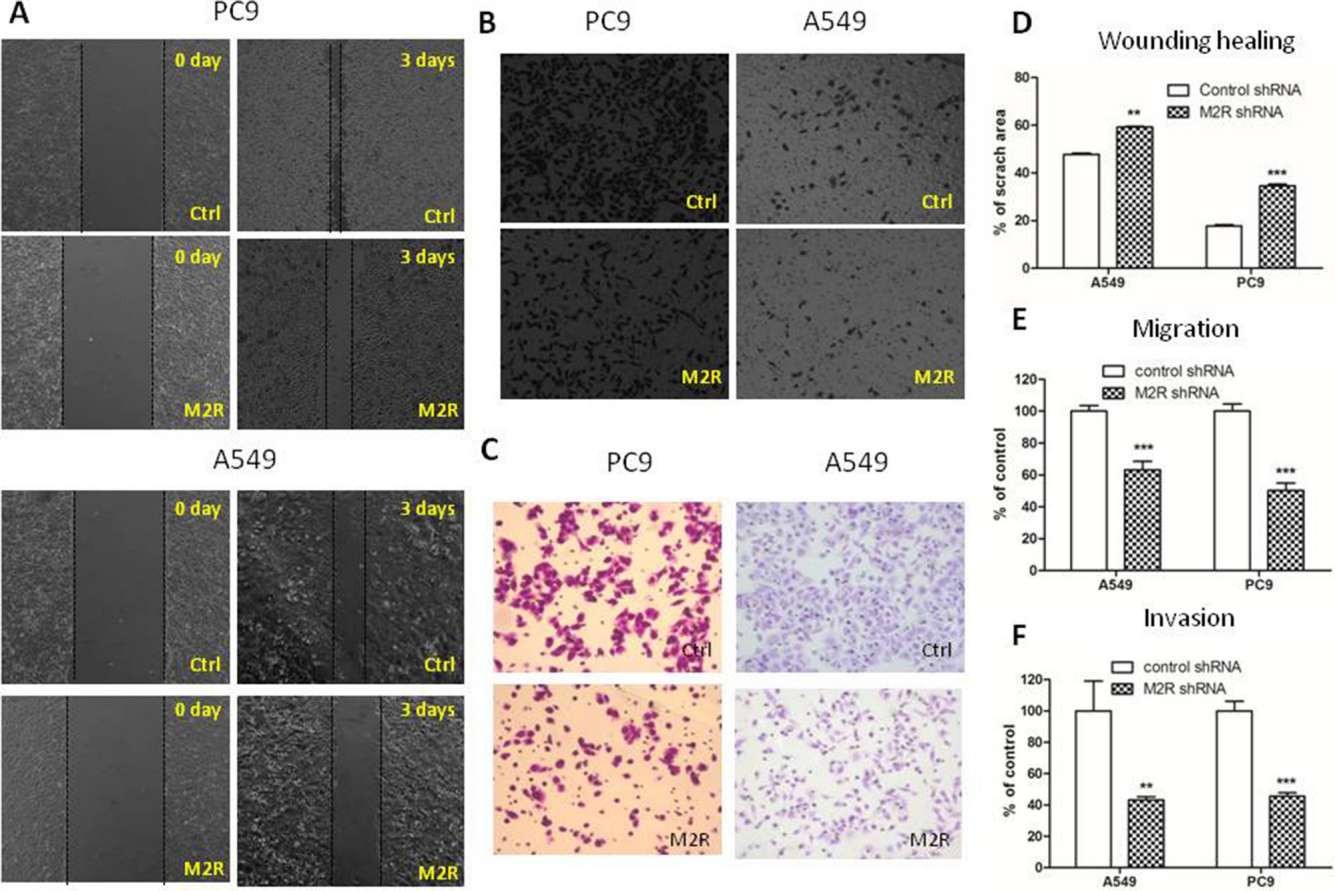
M2R knockdown suppresses the migration and invasion of NSCLC cells Cells were transfected with human M2R shRNA lentiviral particles (M2R) or control shRNA lentiviral particles (Ctrl) and stable cells were selected with puromycin. **A.** Wound healing assay of A549 and PC9 cells stably transfected with M2R shRNA (M2R). Images were taken at 0 and 72 h with a 4× objective lens. The black dotted lines depict the area of wound closure. Transwell migration **B.** and invasion **C.** assay of A549 and PC9 cells stably transfected with M2R shRNA (M2R). After 8 h (B) or 24 h (C) of incubation, cells remaining above the insert membrane were removed by gentle scraping with a sterile cotton swab. Cells that traveled through the insert membrane without (B) or with (C) Matrigel were fixed, stained with crystal violet and counted under light microscopy. Images were taken with a 10× objective lens. **D.** Quantification of wound healing assay. The open wound area was quantified by ImageJ from at least four independent microscopic fields and normalized to the area at time 0. The data are presented as the mean ± SEM. Quantification of transwell migration **E.** and invasion **F.** assay. The number of cells was counted from at least four independent microscopic fields. The data are presented as the means ± SEM and normalized to cell stably transfected with control shRNA (Ctrl). *P < 0.05; **P < 0.01; ***P < 0.001, compared with control.

To test the role of M2R in lung cancer progression, we performed wound healing assay and transwell migration and invasion assay to evaluate *in vitro* migration and invasion ability of NSCLC PC9 and A549 cells. As shown in Figure 1A and 1D, cells treated with 1 μM methoctramine (MT), which is a selective M2R antagonist, rescued much less wounded region at 72 h after wound scratch compared to cells treated with solvent (DMSO). The number of cells migrating through transwell chamber in both non-basement membrane chamber and matrigel-coated chamber was much lower in MT group than that in control group (Figure 1C and 1F). These results suggested that pharmacological blockade of M2R reduced cell migration and invasion. Next, cells were stably transfected with scrambled shRNA (Ctrl) or M2R-specific shRNA (M2R) as described in our previous paper [10] and then subjected to wound healing assay and transwell migration and invasion assay. As shown in Figure 2, knockdown of M2R suppressed the migration (Figure 2A–2E) and invasion (Figure 2C and 2F) of both PC9 and A549 cells, confirming that blocking M2R signaling reduced NSCLC cell migratory and invasive capacities.

### Blocking M2R signaling reversed EMT in NSCLC cells

**Figure 3:**
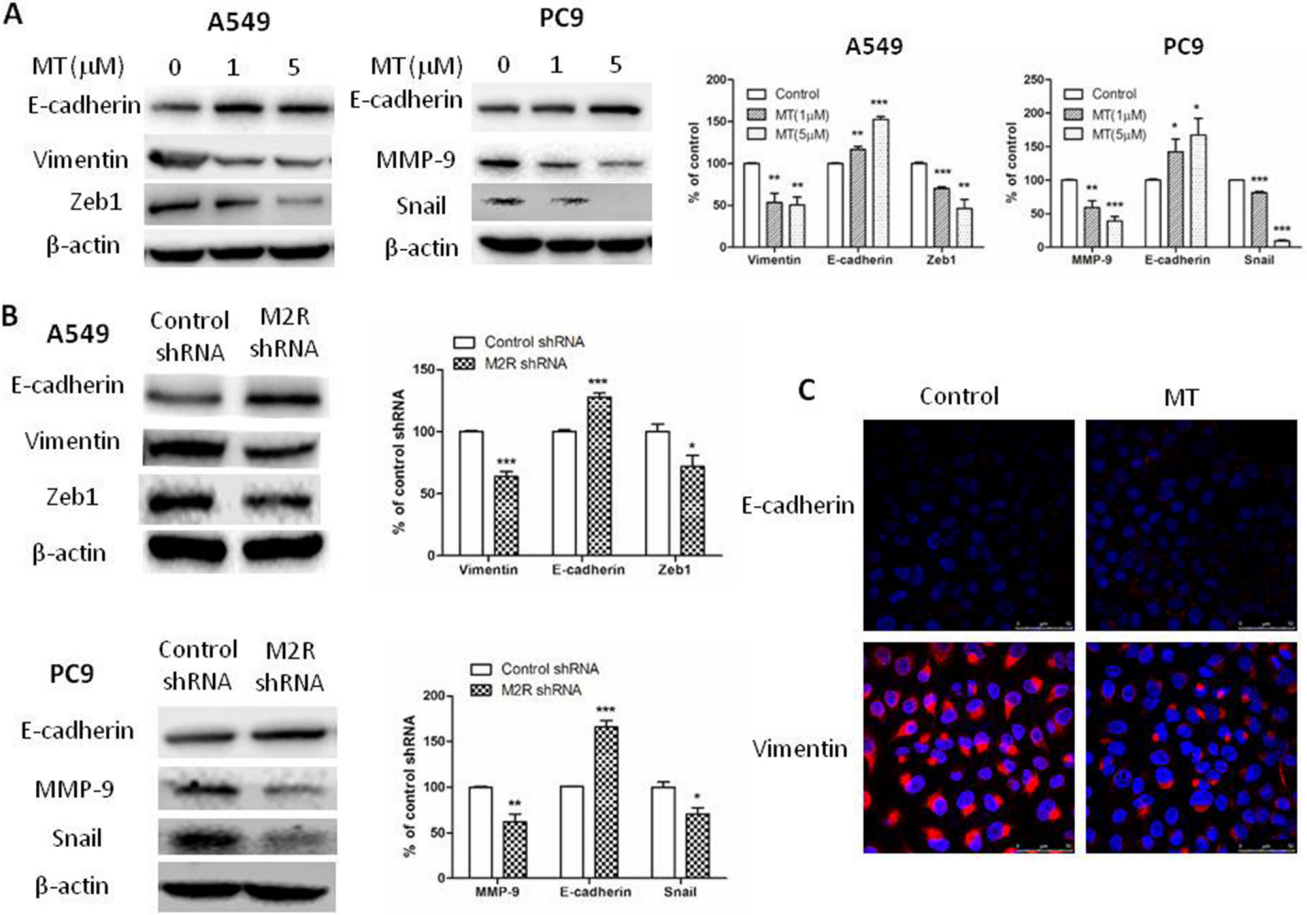
Blocking M2R signaling reverses epithelial-mesenchymal transition (EMT) in NSCLC cells **A.** Cells were treated with the indicated concentrations of methoctramine for 72 h. The expression of EMT-related molecules was measured by Western blot. **B.** Cells were stably transfected with M2R shRNA. The expression of EMT-related molecules was measured by Western blot. β-actin was used as loading control for Western blot. The data are presented as the means ± SEM and normalized to cells treated with solvent (A) or cells stably transfected with control shRNA (B) *P < 0.05; **P < 0.01; ***P < 0.001, compared with control. **C.** A549 cells were treated with 5 μM MT for 72 h and then were stained with fluorescent antibody against E-cadherin or vimentin. Confocal microscopy images showed membrane expression of E-cadherin (red, upper lane) and cytoplasm expression of vimentin (red, lower lane). Nuclei were stained with Hoechst 33342 (blue).

We then examined the expression of EMT-related molecules using Western blot analysis in PC9 and A549 cells. As shown in Figure 3A, epithelial marker E-cadherin was upregulated and mesenchymal markers such as vimentin or matrix metallopeptidase 9 (MMP9) were downregulated in a dose-dependent manner in both cell lines treated with methoctramine. EMT master regulators Snail or ZEB1 were also downregulated by methoctramine treatment in a dose-dependent manner. These results were further confirmed by immunofluorescence experiments in A549 cells. As shown in Figure 3C, E-cadherin was located on the surface of cells while vimentin was located in the cytoplasm of cells. Methoctramine treatment reversed EMT by inducing the expression of E-cadherin and simultaneously repressing the expression of vimentin. Next, we stably transfected cells with scrambled shRNA (Ctrl) or M2R-specific shRNA (M2R) and then examined the expression of EMT-related molecules. As shown in Figure 3B, knockdown of M2R expression increased the expression E-cadherin and decreased the expression of vimentin or MMP9 and EMT master regulators Snail or ZEB1, confirming that blocking M2R signaling reversed EMT in NSCLC. The only source of ligand for M2R in these studies was endogenous ACh released by tumor cells, suggesting that non-neuronal ACh promotes EMT partially through activation of M2R in NSCLC cell lines.

### NF-κB activity was inhibited by M2 antagonist methoctramine in NSCLC cells

**Figure 4:**
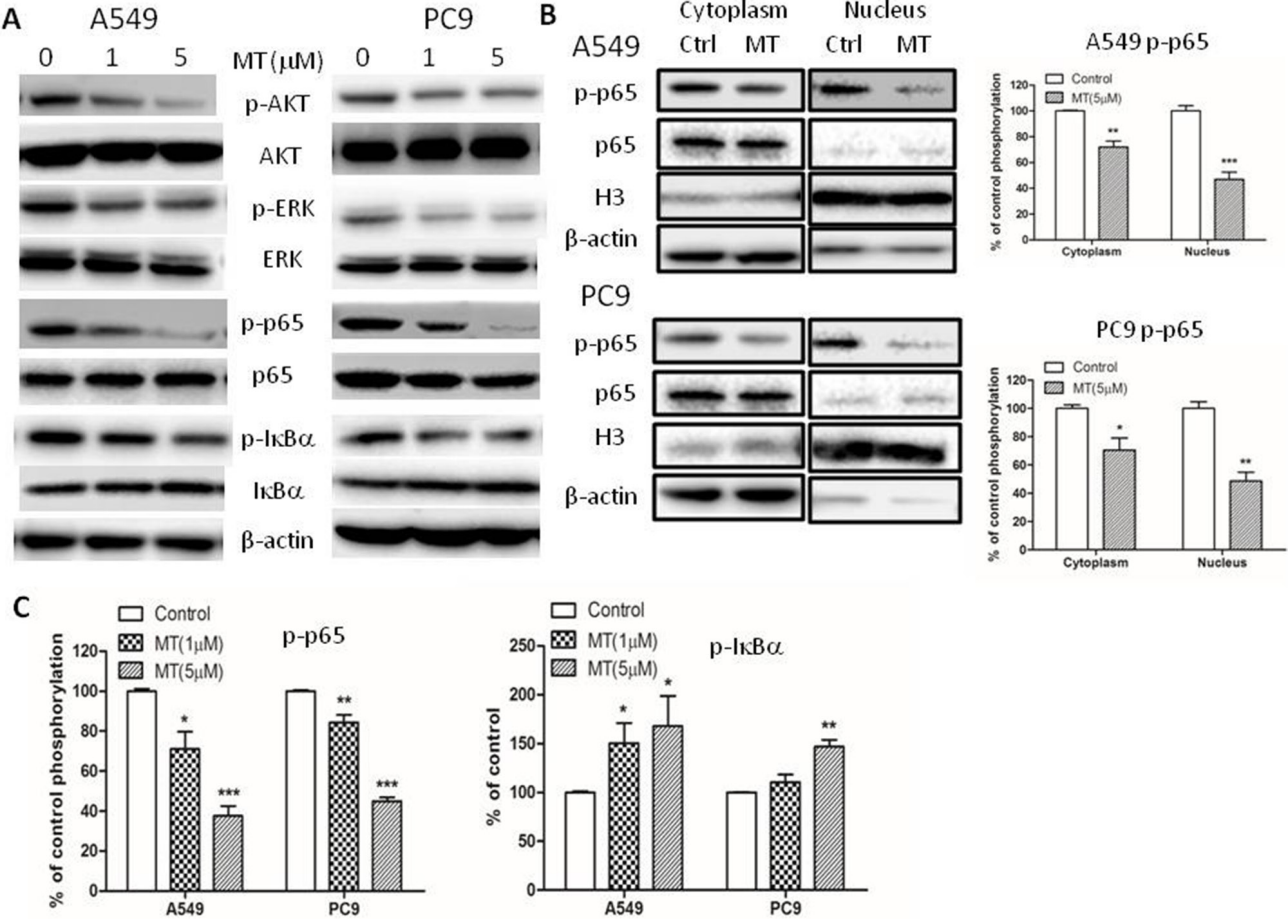
M2R antagonist methoctramine inhibits NF-κB p65 signaling in NSCLC cells Cells were treated with indicated concentrations of methoctramine (MT) of for 72 h. **A.** The expression of p-Akt, Akt, p-ERK, ERK, p-p65, p65, p-IκBα and IκBα in total cellular extracts was measured by Western blot. β-actin was used as loading control. **B.** The expression of p-p65, p65 in cytoplasmic extracts or nuclear extracts was measured by Western blot. β-actin or H3 histone was used as loading control for cytoplasmic extracts or nuclear extracts, respectively. The data are presented as the means ± SEM and normalized to cells treated with solvent. **C.** Quantification of Western blot shown in (A). The data are presented as the means ± SEM and normalized to cells treated with solvent. *P < 0.05; **P < 0.01; ***P < 0.001, compared with control.

Multiple lines of evidence have shown that NF-κB activation is essential for maintenance of an invasive phenotype in cancers. Since blocking M2R signaling reduced migratory and invasive capacities in NSCLC, we explored the effect of methoctramine on the activation of NF-κB p65. Total cellular extracts, cytoplasmic extracts and nuclear extracts of PC9 and A549 cells were prepared for Western blotting analysis following methoctramine treatment for 72 h. As shown in Figure 4 p65 phosphorylation at Ser536 was decreased in total cellular (Figure 4A and 4C), cytoplasmic and nuclear extracts (Figure 4B) in a dose-dependent manner in both cell lines. The phosphorylation of IκBα was decreased while total IκBα was slightly increased in total cellular extracts in a dose-dependent manner (Figure 4A and 4C). Taken together, these results suggested that M2R inhibition by methoctramine suppressed phosphorylation and subsequent degradation of IκBα resulting in less nuclear translocation of p65 and also inhibited phosphorylation of p65, both of which led to inactivation of NF-κB p65 transcriptional activity. However, we failed to detect any significant changes in the levels of p65 expression in the cytoplasm and nucleus possibly due to the sensitivity of Western blot analysis. We also examined the effects of selective M1 and M3 muscarinic receptor antagonists on the activation of NF-κB p65 and found that none of them had effects on NF-κB p65 activation (Supplementary Figure S1).

### Pilocarpine-induced NF-κB activation was inhibited by parthenolide or p65 siRNA and partially suppressed by ERK or Akt inhibitor in NSCLC cells

**Figure 5:**
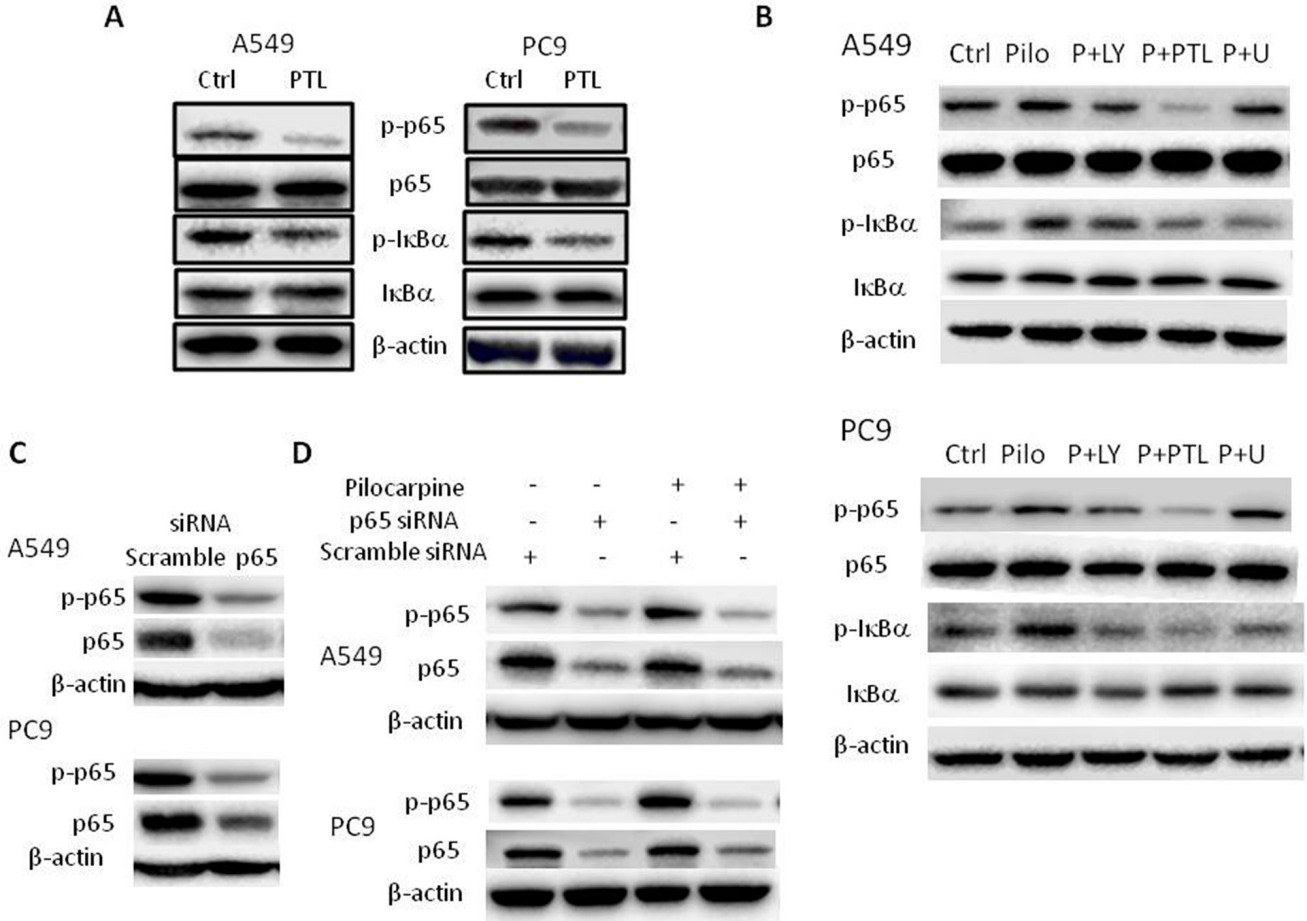
Blocking NF-κB p65 signaling abolishes pilocarpine-induced NF-κB p65 activation in NSCLC cells **A.** Cells were treated with 10 μM parthenolide (PTL) for 24 h. The expression of p-p65, p65, p-IκBα and IκBα was measured by Western blot. **B.** Cells were pretreated with 25 μM LY294002 (LY) or 10 μM PTL or 5 μM U0126 **(U)** for 2 h prior to the stimulation with 10 μM pilocarpine (Pilo) for 24 h. The expression of p-p65, p65, p-IκBα and IκBα was measured by Western blot. **C.** Cells were transfected with p65 siRNA and the expression of p-p65 and p65 was measured by Western blot. **D.** Cells were transfected with p65 siRNA prior to the stimulation with 10 μM pilocarpine for 24 h. The expression of p-p65 and p65 was measured by Western blot. β-actin was used as loading control for Western blot.

Next, we used pharmacological inhibitor parthenolide (PTL) and p65-specific siRNA to inhibit NF-κB signaling. PTL is a selective NF-κB inhibitor and has been used extensively to inhibit NF-κB activation. As shown in Figure 5A, PTL decreased the phosphorylation of NF-κB p65 and IκBα, resulting in the inhibition of NF-κB signaling in both cell lines. We also used p65-specific siRNA to knockdown the expression of NF-κB p65 and as shown in Figure 5C, siRNA-mediated p65 knockdown significantly decreased the levels of p65 and p-p65. Then, exogenous muscarinic receptor agonist pilocarpine was used to activate M2R signaling. Our results showed that pilocarpine increased the phosphorylation of NF-κB p65 and IκBα, resulting in NF-κB activation in both cell lines (Figure 5B). Pretreatment of cells with PTL or siRNA p65 completely abolished pilocarpine-induced NF-κB p65 activation (Figure 5B and 5D). Combined with above results that M2R inhibition by methoctramine suppressed NF-κB activity (Figure 4), our data strongly suggested for the first time that NF-κB is the downstream effector of M2R signaling.

Growing evidence has shown that PI3K/Akt and MAPK ERK could directly phosphorylate and activate IKK or phosphorylate p65, either of which leads to NF-κB activation [32–38]. In Figure 4, pilocarpine has been shown to induce the phosphorylation of Akt and ERK and methoctramine to inhibit the phosphorylation of Akt and ERK, suggesting that Akt or ERK could be the molecular link between M2R and NF-κB. We further investigated the effects of inhibiting Akt or ERK on pilocarpine-induced NF-κB activation, using their respective inhibitor, LY294002 (LY) and U0126 (U). As shown in Figure 5B, pilocarpine-induced phosphorylation of NF-κB p65 and IκBα was completely abolished by PTL treatment. Moreover, pilocarpine-induced phosphorylation of IκBα was completed ablated in PC9 cells pretreated with LY or U and in A549 cells pretreated with U, while it was only partially ablated in A549 cells pretreated with LY, suggesting that Akt and ERK could activate NF-κB by stimulating phosphorylation and subsequent degradation of IκBα. Phosphorylation of p65 induced by pilocarpine was partially ablated by LY or U in A549 cells and LY in PC9 cells, suggesting that Akt could also directly phosphorylate p65 in both cell lines while ERK could only in A549 cells. Taken together, M2R activation could activate downstream NF-κB signaling partially through PI3K/Akt and ERK in NSCLC.

### Inhibition of NF-κB signaling suppressed NSCLC cell migration and invasion under basal and pilocarpine-induced conditions

**Figure 6:**
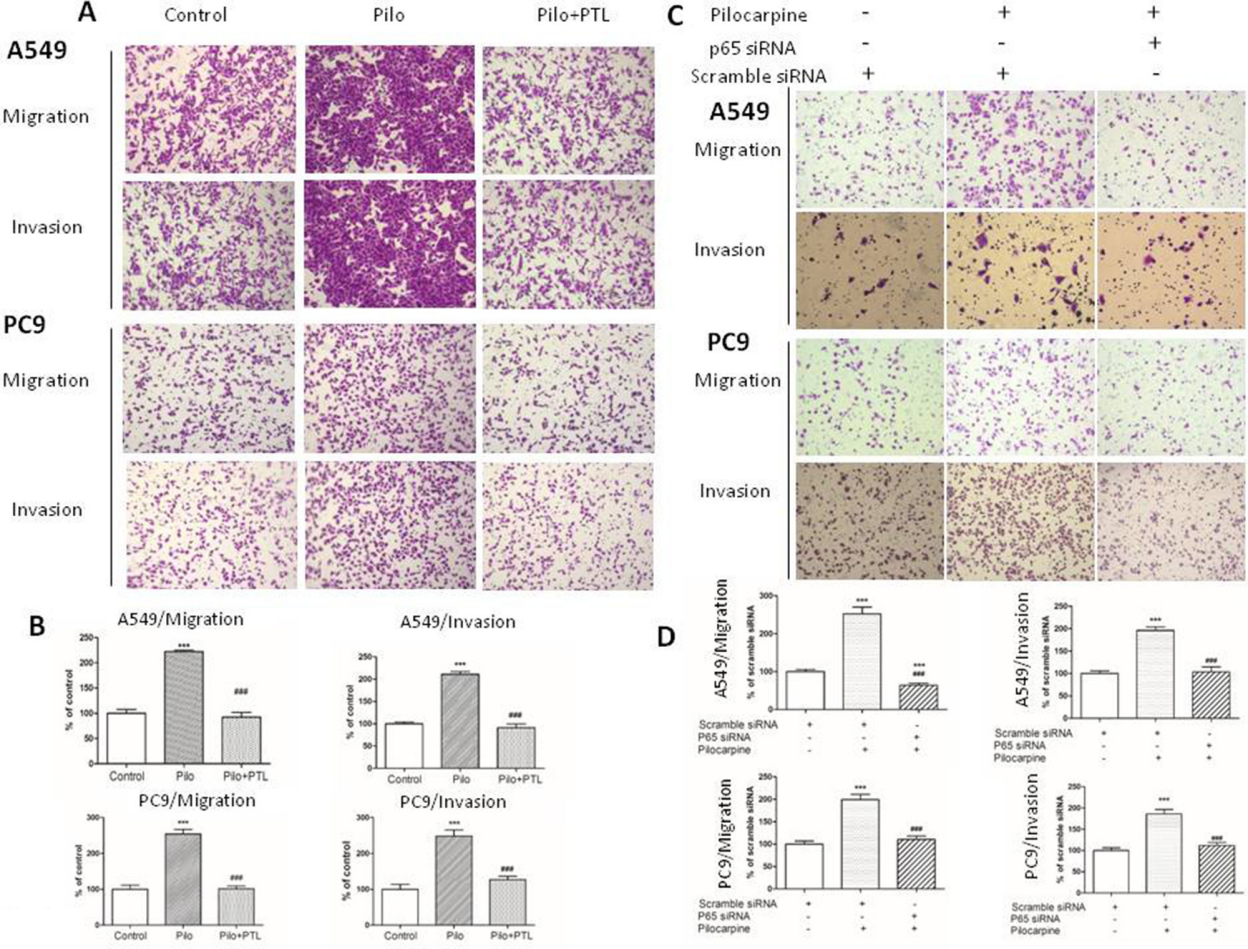
Blocking NF-κB p65 signaling suppresses NSCLC cell migration and invasion induced by pilocarpine **A.** Transwell migration and invasion assay of A549 and PC9 cells pretreated with 10 μM PTL for 2 h prior to the stimulation with 10 μM pilocarpine for 24 h. **C.** Transwell migration and invasion assay of A549 and PC9 cells transfected with p65 siRNA prior to the stimulation with 10 μM pilocarpine for 24 h. **B.** and **D.** Quantification of transwell assay shown in (A) and (C), respectively. The number of cells was counted from at least four independent microscopic fields. The data are presented as the means ± SEM and normalized to cells treated with solvent (A) or cells transfected with control siRNA (C) *P < 0.05; **P < 0.01; ***P < 0.001, compared with control. ^#^, *P* < 0.05; ^##^, *P* < 0.01; ^###^, P < 0.001, compared with pilocarpine.

To further demonstrate that NF-κB signaling pathway is essential for the effects of non-neuronal ACh, we used PTL (Figure 6A and 6B) and p65 siRNA (Figure 6C and 6D) to inhibit NF-κB signaling and then performed transwell migration and invasion assay. As shown in Figure 6A and 6C, pilocarpine increased migratory and invasive capacities in both cell lines. Pretreatment of PTL or siRNA-mediated p65 knockdown completely abolished pilocarpine-induced upregulation of migration and invasion. These results suggested that NF-κB activation is required for enhanced migratory and invasive capacities induced by muscarinic activation.

### Inhibition of NF-κB signaling reversed EMT under basal and pilocarpine-induced conditions in NSCLC cells

**Figure 7:**
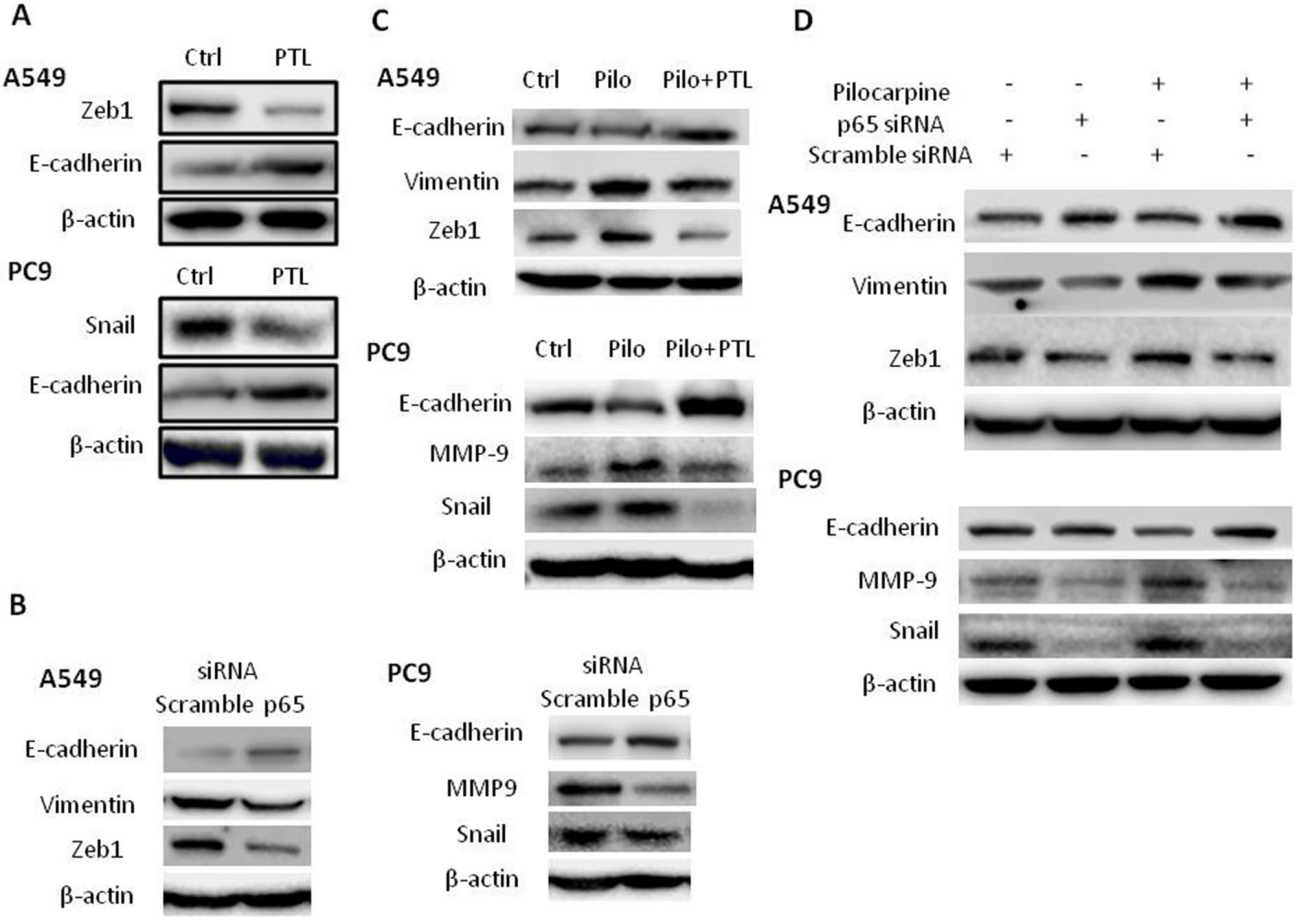
Blocking NF-κB p65 signaling reverses EMT under basal and pilocarpine-induced conditions **A.** Cells were treated with 10 μM PTL for 24 h. **B.** Cells were transfected with p65 siRNA. **C.** Cells were treated with 10 μM PTL for 2 h prior to the stimulation with 10 μM pilocarpine (Pilo) for 24 h. **D.** Cells were transfected with p65 siRNA prior to the stimulation with 10 μM pilocarpine for 24 h. The expression of EMT-related molecules was measured by Western blot. β-actin was used as loading control for Western blot.

Next, we investigated the roles of NF-κB activation in the regulation of EMT induced by endogenous ACh or exogenous muscarinic agonist pilocarpine. As shown in Figure 7A, the E-cadherin levels were increased while the Snail or ZEB1 levels were decreased in PTL-treated group compared to control group in both cell lines, suggesting PTL reversed EMT in NSCLC. Knockdown of p65 expression by siRNA also reversed EMT by decreasing the levels of Vimentin/MMP9 and Zeb1/Snail (Figure 7B). Pretreatment of cells with PTL (Figure 7C) or p65 siRNA (Figure 7D) also reversed EMT induced by pilocarpine. Taken together, these results indicated that NF-κB activation plays an essential role in EMT induced by non-neuronal ACh through muscarinic activation in NSCLC.

## DISCUSSION

Lung cancer cells can synthesize and secret ACh which in turn acts as an autoparacrine growth factor to stimulate tumor growth via activation of nicotinic and muscarinic acetylcholine (ACh) receptors (nAChR, mAChR). nAChR belongs to the family of ligand-gated ion channels. Extensive studies have shown that activation of nAChR leads to channel opening and influx of calcium which activates signaling cascades involved in cell proliferation, apoptotic inhibition, migration and angiogenesis [39–41]. mAChR belongs to G-protein coupled receptors family and five subtypes of mAChRs (M1-M5) have been identified. M1, M3, M5 receptors are coupled with Gq proteins, while M2 and M4 receptors are coupled with Gi/Go proteins. However, the roles of mAChR in carcinogenesis and tumor progression are not fully understood. Our previous work has shown that blocking M2R signaling can suppress EMT, which is the key process in cancer progression and metastasis. This paper extends these previous results to delineate molecular mechanisms whereby blocking M2R signaling suppresses EMT, which may provide potential therapeutic targets to delay lung cancer progression.

EMT is associated with functional changes relevant to metastasis, i.e. increased migratory and invasive abilities. During EMT, epithelial cells lose their epithelial polarity and cell-cell contacts and gain mesenchymal phenotypes with increased migratory and invasive abilities. A hallmark of EMT is the loss of E-cadherin, which is mediated by a network of transcription factors including the Snail, Zeb and Twist families that target the CDH1 gene promoter. EMT can be induced by intracellular cues, such as NF-κB signaling or by extracellular growth factors. Our previous study and many others have indicated that non-neuronal ACh, as an autoparacrine growth factor could induce EMT through activation of α7 nAChR and M2R [10, 13]. However, the underlying mechanism remains unclear.

Many studies have reported that NF-κB activation is closely involved in the induction and maintenance of EMT [25–31]. NF-κB not only regulates the transcription of EMT master regulator genes, such as Snail, Slug and ZEB1, which repress the epithelial phenotype by downregulating E-cadherin but also controls the mesenchymal genes, such as VIM. In this paper, we have shown that M2R selective antagonist methoctramine inhibits phosphorylation of IκBα and p65, resulting in the inactivation of NF-κB signaling in NSCLC. However, antagonizing M1 or M3 mAChR does not inhibit NF-κB signaling. Conversely, exogenous mAChR agonist pilocarpine activates NF-κB signaling. Furthermore, pharmacological inhibition or genetic disruption of NF-κB p65 reverses EMT as well as reduces migratory and invasive ability in NSCLC. Take together, our results demonstrate for the first time that non-neuronal ACh could induce EMT through activation of M2R and downstream NF-κB signaling.

NF-κB signaling could be directly activated by PI3K/Akt or MAPK ERK pathways [32–38]. Our data show that mAChR agonist, pilocarpine stimulates phosphorylation of ERK and Akt while M2R inhibition decreases ERK and Akt phosphorylation. More importantly, inhibition of ERK or Akt partially abolishes pilocarpine-induced NF-κB p65 activation, suggesting that non-neuronal ACh may activate M2R and downstream ERK and Akt to enhance NF-κB signaling.

Dysfunction of the non-neuronal cholinergic system is involved in the pathogenesis of cancer. In lung cancer, cholinergic signaling is markedly upregulated which presents potential therapeutic targets [5–10]. Our results demonstrate for the first time that M2R/ERK/Akt/NF-κB signaling axis plays an important role in lung cancer progression associated with non-neuronal cholinergic system. NF-κB is also at the center of multiple signaling pathways that promote tumor invasiveness. Inhibition of NF-κB could attenuate the progression and metastasis of cancer. Given that NF-κB also plays a pivotal role in immune system, the challenge is how to target it selectively in cancer. Blocking the nonredundant, cancer-specific downstream effectors of NF-κB will hopefully provide new strategies for targeting NF-κB.

## MATERIALS AND METHODS

### Reagents

Pilocarpine and methoctramine were purchased from Sigma-Aldrich (St. Louis, MO, USA). Parthenolide, LY294002 and U0126 were purchase from Selleck (Shanghai, China). Antibodies used for Western blotting were purchased from Cell Signaling Technology (Danvers, MA, USA) for Erk, phospho-p42/44 Erk (Thr202/ Tyr204), Akt, phospho-Akt (Ser473), E-cadherin, vimentin, MMP9, Snail, Zeb1, phospho-NF-κB p65 (Ser536), NF-κB p65, IκBα, phosphor-IκBα (Ser32)and β-actin.

### NSCLC cell culture

Human NSCLC A549 cells were purchased from the Cell Bank of Type Culture Collection of the Chinese Academy of Sciences (Shanghai, China) and PC9 cells were gifts from Dr. Qianggang Dong in Shanghai Cancer Institute, Shanghai Jiao Tong University School of Medicine. Both cell lines are human lung adenocarcinoma cell lines and were routinely cultured in DMEM (Invitrogen, Carlsbad, CA, USA) supplemented with 10% fetal bovine serum (Invitrogen), 100 μg/ml penicillin and 100 U/ml streptomycin sulfate in a humidified incubator at 37°C with 5% CO_2_.

### Western blot

Total cellular extracts were obtained by lysing cell pellets in RIPA buffer (50 mM Tris, pH7.4, 150 mM NaCl, 1% Triton X-100, 1% sodium deoxycholate, 0.1% SDS), while cytoplasmic extracts and nuclear extracts were prepared by using Nuclear and Cytoplasmic Protein Extraction Kit (Beyotime, Haimen, China) according to the manufacturer's instruction. All lysis buffers were supplemented with protease inhibitor (Sigma-Aldrich) and phosphatase inhibitors (Sangon Biotech, Shanghai, China). Equal amounts of proteins were separated on 10% SDS-PAGE and then transferred to PVDF membranes (Millipore, Billerica, MA, USA). Membranes were blocked with PBS buffer containing 5% non-fat milk and 0.1% Tween 20 (PBST) and then incubated with primary antibodies (1:500–1:1000) overnight at 4°C. After being washed three times with PBST, membranes were incubated with peroxidase-conjugated secondary antibodies (1:3000) for 1 h and then washed with PBST again and developed with ECL (Pierce, Rockford, IL, USA). β-actin was used as an internal control for loading control. Relative quantities of proteins in Western blotting were analyzed using Image J Software (US National Institutes of Health).

### siRNA transfection and lentivirus-mediated transduction of shRNA

NF-κB p65 knockdown was performed by transfecting cells with NF-κB p65 siRNA (GenePharma, Shanghai, China) according to the manufacturer's instructions. NF-κB p65 siRNA sequences are as following: 5′-GCCCTATCCCTTTACGTCATT-3′ and 5′-TGACGTAAAGGGATAGGGCTT-3′. The cells were subjected to further experiments after 48 h after transfection.

M2R shRNA lentiviral particles were purchased from Santa Cruz Biotechnology and the lentvirus transduction was performed as described previously [10]. Cells with stable integration of shRNA constructs were selected with puromycin (5 μg/ml).

### Transwell migration and invasion assay

Migration assay was performed in 24-well inserts (8-μm pore size; Corning Inc, Corning, NY, USA) and cell invasion assay was performed in 24-well Matrigel invasion inserts (8-μm pore size; Corning Inc, Corning, NY, USA) according to manufacturer's instructions. 3 × 10^4^ cells in serum-free DMEM were seeded in the upper chamber of the insert and DMEM containing 10% fetal bovine serum was added to the lower chamber. Cells were incubated at 37°C in 5% CO_2_ for 8 h (for migration) or 1 day (for invasion). Nonmigrated cells were scraped from the upper surface of the membrane with a cotton swab, and migrated cells remaining on the bottom surface were fixed with 10% paraformaldehyde for 20 min, stained with a 0.1% crystal violet solution for 2 hours, and then photographed and counted under a microscopy from at least four randomly selected fields.

### Wound healing assay

Cells were allowed to grow to 90% confluency in 6-well flat-bottomed plates and serum starved for 24 h in medium containing 1% FBS. After aspirating the medium, the monolayer was scratched using a 200 μL pipette tip. The cells were washed with PBS to remove detached cells and then incubated in medium containing 5% FBS with or without 5 μM methoctramine. The scratched areas were photographed at 0 and 48 h after scratching using microscopy. Cell migration was calculated as percentages of open wound area to the initial blank areas containing no cells using ImageJ software. The values are the means of three independent experiments.

### Immunofluorescence and confocal microscopy

Cells were fixed in 4% paraformaldehyde, permeabilized in 0.2% Triton X-100, blocked in 1% BSA, and then stained with antibody (1:100) at 4°C overnight. After being washed three times with PBS, cells were incubated with FITC-conjugated secondary antibodies (1:100) for 1 h, and then washed with PBS again and incubated with Hoest 33342 for nuclear counterstaining. Cells were visualized with a Zeiss LSM710 (Zeiss, Thornwood, NY, USA) confocal microscope.

### Statistical analysis

All data are presented as the mean±SEM. Statistical analysis was conducted using GraphPad Prism 5.0 software (La Jolla, CA, USA). Differences between groups were examined using Student's *t*-test. Differences were considered significant if P value was less than 0.05.

## SUPPLEMENTARY FIGURE



## References

[1] Beckmann J, Lips KS (2013). The non-neuronal cholinergic system in health and disease. Pharmacology.

[2] Proskocil BJ, Sekhon HS, Jia Y, Savchenko V, Blakely RD, Lindstrom J, Spindel ER (2004). Acetylcholine is an autocrine or paracrine hormone synthesized and secreted by airway bronchial epithelial cells. Endocrinology.

[3] Song P, Sekhon HS, Proskocil B, Blusztajn JK, Mark GP, Spindel ER (2003). Synthesis of acetylcholine by lung cancer. Life Sci.

[4] Wessler I, Kirkpatrick CJ (2008). Acetylcholine beyond neurons: the non-neuronal cholinergic system in humans. Br J Pharmacol.

[5] Song P, Sekhon HS, Jia Y, Keller JA, Blusztajn JK, Mark GP, Spindel ER (2003). Acetylcholine is synthesized by and acts as an autocrine growth factor for small cell lung carcinoma. Cancer Res.

[6] Paleari L, Catassi A, Ciarlo M, Cavalieri Z, Bruzzo C, Servent D, Cesario A, Chessa L, Cilli M, Piccardi F, Granone P, Russo P (2008). Role of alpha7-nicotinic acetylcholine receptor in human non-small cell lung cancer proliferation. Cell Prolif.

[7] Song P, Sekhon HS, Lu A, Arredondo J, Sauer D, Gravett C, Mark GP, Grando SA, Spindel ER (2007). M3 muscarinic receptor antagonists inhibit small cell lung carcinoma growth and mitogen-activated protein kinase phosphorylation induced by acetylcholine secretion. Cancer Res.

[8] Hua N, Wei X, Liu X, Ma X, He X, Zhuo R, Zhao Z, Wang L, Yan H, Zhong B, Zheng J (2012). A novel muscarinic antagonist R2HBJJ inhibits non-small cell lung cancer cell growth and arrests the cell cycle in G0/G1. PLoS One.

[9] Song P, Sekhon HS, Fu XW, Maier M, Jia Y, Duan J, Proskosil BJ, Gravett C, Lindstrom J, Mark GP, Saha S, Spindel ER (2008). Activated cholinergic signaling provides a target in squamous cell lung carcinoma. Cancer Res.

[10] Zhao Q, Gu X, Zhang C, Lu Q, Chen H, Xu L (2015). Blocking M2 muscarinic receptor signaling inhibits tumor growth and reverses epithelial-mesenchymal transition (EMT) in non-small cell lung cancer (NSCLC). Cancer Biol Ther.

[11] Tsai JH, Yang J (2013). Epithelial-mesenchymal plasticity in carcinoma metastasis. Genes Dev.

[12] Lamouille S, Xu J, Derynck R (2014). Molecular mechanisms of epithelial-mesenchymal transition. Nat Rev Mol Cell Biol.

[13] Dasgupta P, Rizwani W, Pillai S, Kinkade R, Kovacs M, Rastogi S, Banerjee S, Carless M, Kim E, Coppola D, Haura E, Chellappan S (2009). Nicotine induces cell proliferation, invasion and epithelial-mesenchymal transition in a variety of human cancer cell lines. Int J Cancer.

[14] Davis R, Rizwani W, Banerjee S, Kovacs M, Haura E, Coppola D, Chellappan S (2009). Nicotine promotes tumor growth and metastasis in mouse models of lung cancer. PLoS One.

[15] Wu SQ, Lv YE, Lin BH, Luo LM, Lv SL, Bi AH, Jia YS (2013). Silencing of periostin inhibits nicotine-mediated tumor cell growth and epithelial-mesenchymal transition in lung cancer cells. Mol Med Rep.

[16] De Craene B, Berx G (2013). Regulatory networks defining EMT during cancer initiation and progression. Nat Rev Cancer.

[17] Zheng H, Kang Y (2014). Multilayer control of the EMT master regulators. Oncogene.

[18] Min C, Eddy SF, Sherr DH, Sonenshein GE (2008). NF-kappaB and epithelial to mesenchymal transition of cancer. J Cell Biochem.

[19] Viatour P, Merville MP, Bours V, Chariot A (2005). Phosphorylation of NF-kappaB and IkappaB proteins: implications in cancer and inflammation. Trends Biochem Sci.

[20] Hu J, Nakano H, Sakurai H, Colburn NH (2004). Insufficient p65 phosphorylation at S536 specifically contributes to the lack of NF-kappaB activation and transformation in resistant JB6 cells. Carcinogenesis.

[21] Karin M, Greten FR (2005). NF-kappaB: linking inflammation and immunity to cancer development and progression. Nat Rev Immunol.

[22] Karin M (2006). Nuclear factor-kappaB in cancer development and progression. Nature.

[23] Fujioka S, Sclabas GM, Schmidt C, Frederick WA, Dong QG, Abbruzzese JL, Evans DB, Baker C, Chiao PJ (2003). Function of nuclear factor kappaB in pancreatic cancer metastasis. Clin Cancer Res.

[24] Fujioka S, Sclabas GM, Schmidt C, Niu J, Frederick WA, Dong QG, Abbruzzese JL, Evans DB, Baker C, Chiao PJ (2003). Inhibition of constitutive NF-kappa B activity by I kappa B alpha M suppresses tumorigenesis. Oncogene.

[25] Huber MA, Azoitei N, Baumann B, Grünert S, Sommer A, Pehamberger H, Kraut N, Beug H, Wirth T (2004). NF-kappaB is essential for epithelial-mesenchymal transition and metastasis in a model of breast cancer progression. J Clin Invest.

[26] Kumar M, Allison DF, Baranova NN, Wamsley JJ, Katz AJ, Bekiranov S, Jones DR, Mayo MW (2013). NF-κ;B regulates mesenchymal transition for the induction of non-small cell lung cancer initiating cells. PLoS One.

[27] Wamsley JJ, Kumar M, Allison DF, Clift SH, Holzknecht CM, Szymura SJ, Hoang SA, Xu X, Moskaluk CA, Jones DR, Bekiranov S, Mayo MW (2015). Activin upregulation by NF-κ;B is required to maintain mesenchymal features of cancer stem-like cells in non-small cell lung cancer. Cancer Res.

[28] Maier HJ, Schmidt-Strassburger U, Huber MA, Wiedemann EM, Beug H, Wirth T (2010). NF-kappaB promotes epithelial-mesenchymal transition, migration and invasion of pancreatic carcinoma cells. Cancer Lett.

[29] Song FN, Duan M, Liu LZ, Wang ZC, Shi JY, Yang LX, Zhou J, Fan J, Gao Q, Wang XY (2014). RANKL promotes migration and invasion of hepatocellular carcinoma cells via NF-κ;B-mediated epithelial-mesenchymal transition. PLoS One.

[30] Julien S, Puig I, Caretti E, Bonaventure J, Nelles L, van Roy F, Dargemont C, de Herreros AG, Bellacosa A, Larue L (2007). Activation of NF-kappaB by Akt upregulates Snail expression and induces epithelium mesenchyme transition. Oncogene.

[31] Maier HJ, Schmidt-Strassburger U, Huber MA, Wiedemann EM, Beug H, Wirth T (2010). NF-kappaB promotes epithelial-mesenchymal transition, migration and invasion of pancreatic carcinoma cells. Cancer Lett.

[32] Chen BC, Lin WW (2001). PKC- and ERK-dependent activation of I kappa B kinase by lipopolysaccharide in macrophages: enhancement by P2Y receptor-mediated CaMK activation. Br J Pharmacol.

[33] Jiang B, Xu S, Hou X, Pimentel DR, Brecher P, Cohen RA (2004). Temporal control of NF-kappaB activation by ERK differentially regulates interleukin-1beta-induced gene expression. J Biol Chem.

[34] Tyagi N, Bhardwaj A, Singh AP, McClellan S, Carter JE, Singh S (2014). p-21 activated kinase 4 promotes proliferation and survival of pancreatic cancer cells through AKT- and ERK-dependent activation of NF-κ;B pathway. Oncotarget.

[35] Arora S, Bhardwaj A, Singh S, Srivastava SK, McClellan S, Nirodi CS, Piazza GA, Grizzle WE, Owen LB, Singh AP (2013). An undesired effect of chemotherapy: gemcitabine promotes pancreatic cancer cell invasiveness through reactive oxygen species-dependent, nuclear factor kappaB- and hypoxia-inducible factor 1alpha-mediated up-regulation of CXCR4. J Biol Chem.

[36] Martelli AM, Nyåkern M, Tabellini G, Bortul R, Tazzari PL, Evangelisti C, Cocco L (2006). Phosphoinositide 3-kinase/Akt signaling pathway and its therapeutical implications for human acute myeloid leukemia. Leukemia.

[37] Hu J, Nakano H, Sakurai H, Colburn NH (2004). Insufficient p65 phosphorylation at S536 specifically contributes to the lack of NF-kappaB activation and transformation in resistant JB6 cells. Carcinogenesis.

[38] Guo C, Stark GR (2011). FER tyrosine kinase (FER) overexpression mediates resistance to quinacrine through EGF-dependent activation of NF-kappaB. Proc Natl Acad Sci U S A.

[39] Improgo MR, Tapper AR (2011). Nicotinic acetylcholine receptor-mediated mechanisms in lung cancer. Biochem Pharmacol.

[40] Grando SA (2014). Connections of nicotine to cancer. Nat Rev Cancer.

[41] Russo P, Del Bufalo A, Milic M, Salinaro G, Fini M, Cesario A (2014). Cholinergic receptors as target for cancer therapy in a systems medicine perspective. Curr Mol Med.

