# Supplementary material for: Inactivation of M2 AChR/NF-κB signaling axis reverses epithelial-mesenchymal transition (EMT) and suppresses migration and invasion in non-small cell lung cancer (NSCLC)
Source: Oncotarget. 2015 Aug 20;6(30):29335–46. doi: 10.18632/oncotarget.5004 (PMC4745730; doi:10.18632/oncotarget.5004)
Supplement: Supplementary file 1 [file oncotarget-06-29335-s001.pdf]

SUPPLEMENTARY FIGURE

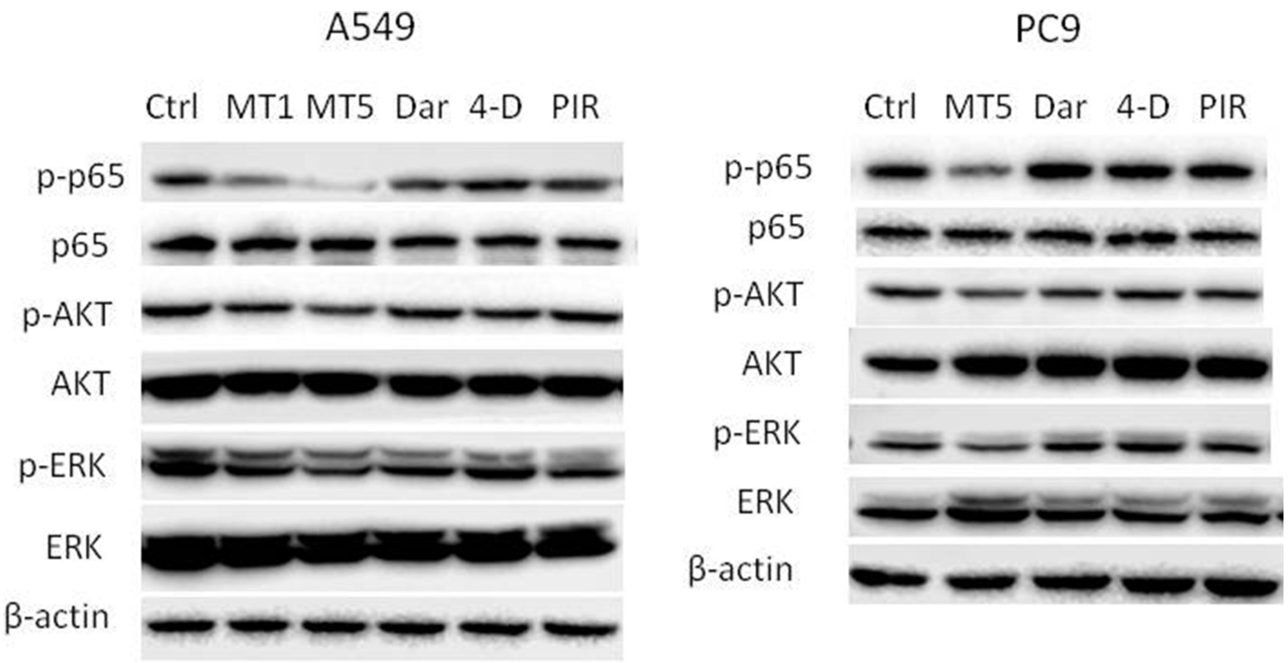

Supplementary Figure S1: M1 and M3 mAChR antagonists have no effects on NF-κB p65 signaling in NSCLC cells. Cells were treated with MT (1 µM or 5 µM) or darifenacin (Dar, 5 µM) or 4-DAMP (4-D, 5 µM) or pirenzepine (PIR, 5 µM) for 72 h. The expression of p-Akt, Akt, p-ERK, ERK, p-p65 and p65 was measured by Western blot. β-actin was used as loading control for Western blot.
